# Central and mid-peripheral corneal astigmatism in an elderly population: a retrospective analysis of Scheimpflug topography results

**DOI:** 10.1038/s41598-021-81772-w

**Published:** 2021-04-12

**Authors:** Grzegorz Łabuz, Dorottya Varadi, Ramin Khoramnia, Gerd U. Auffarth

**Affiliations:** David J Apple Center for Vision Research, Department of Ophthalmology, University Hospital Heidelberg, INF 400, 69120 Heidelberg, Germany

**Keywords:** Epidemiology, Translational research

## Abstract

Implantation of toric intraocular lenses (IOLs) has become standard in the correction of corneal astigmatism. The IOL selection is based on keratometric measurements of the central cornea. However, mid-peripheral corneal changes may yield suboptimal correction in patients with larger pupils. This study retrospectively analyzed corneal topography data collected using a Scheimpflug device during routine clinical examinations. Of 11,953 patients, 641 met the inclusion criteria. Total corneal astigmatism was compared between five concentric zones (2–6 mm) using vector analysis. The absolute difference between astigmatism at 2 mm and 6 mm was 0.30 D (− 0.36 to 0.64), which decreased to 0.10 D (0 to 0.20) between the 5- and 6-mm zone. With-the-rule astigmatism was the most prevalent (53%), 34% had against-the-rule (ATR), and 13% had oblique. The decrease of the cylinder power with the diameter differed significantly between the three types, with ATR and oblique astigmatism being associated with the steepest change. Patients with high corneal astigmatism tend to demonstrate larger differences between the center and mid-periphery than those with low and moderate astigmatism. In conclusion, we demonstrated that central corneal astigmatism differs from that measured at the mid-periphery and that a larger difference was found in patients with ATR, oblique and high astigmatism.

## Introduction

The aim of modern cataract surgery and refractive lens exchange is to provide patients with excellent quality of vision primarily at far but also intermediate and near distances. In order to achieve the best postoperative outcome, apart from factors like previous surgeries or ocular comorbidities, precise biometry is of paramount importance, which includes accurate measurements of the axial length, the anterior chamber depth, and keratometry. Postoperative vision may still be limited by higher-order aberrations and light scattering (straylight)^1,2^, most importantly, however, by residual refractive error and uncorrected cylinder^3^. Thus, correcting even low astigmatism has the potential to improve visual function^4–7^.

Large-population studies have shown astigmatism equal to or greater than 1 D affects from 34.8 to 42% of patients scheduled for cataract surgery^8,9^. Besides the increase of the magnitude of astigmatism^10^, the type of corneal astigmatism changes significantly with age. Wakefield et al. and Asano et al. reported that with-the-rule (WTR) astigmatism, the steepest meridian is oriented vertically, converts with age to against-the-rule (ATR) when the steepest median is closer to the horizontal axis^10,11^. Oblique astigmatism is the least common form, which delineates corneas that do not fall into WTR or ATR category^10,11^. Although the anterior corneal surface yields the most substantial contribution to the refractive (cylinder) power of the eye, the posterior cornea can also contribute to total astigmatism. It is particularly important in the calculation of toric intraocular lenses (IOLs). Koch et al. demonstrated that the posterior corneal surface lowers total astigmatism in eyes with WTR astigmatism, but this effect is reversed in ATR cases^12^.

In toric IOL implantation, the selection of cylinder power is based on corneal topography (both anterior and posterior) taken in the central area. However, flattening of the peripheral cornea may result in a decrease of the cylinder power with increasing distance from the center^13,14^. The conventional toric IOLs may not be able to correct peripheral astigmatism adequately. This, potentially, could have an effect on the visual quality in patients with large pupils under scotopic conditions^15^. Thus, it is essential to determine the extent of mid-peripheral astigmatism changes in a large-cohort of cataract patients.

This study aimed to assess the distribution of the total cylinder power measured in five corneal zones (from 2 to 6 mm) in an elderly astigmatic population.

## Methods

**Figure 1:**
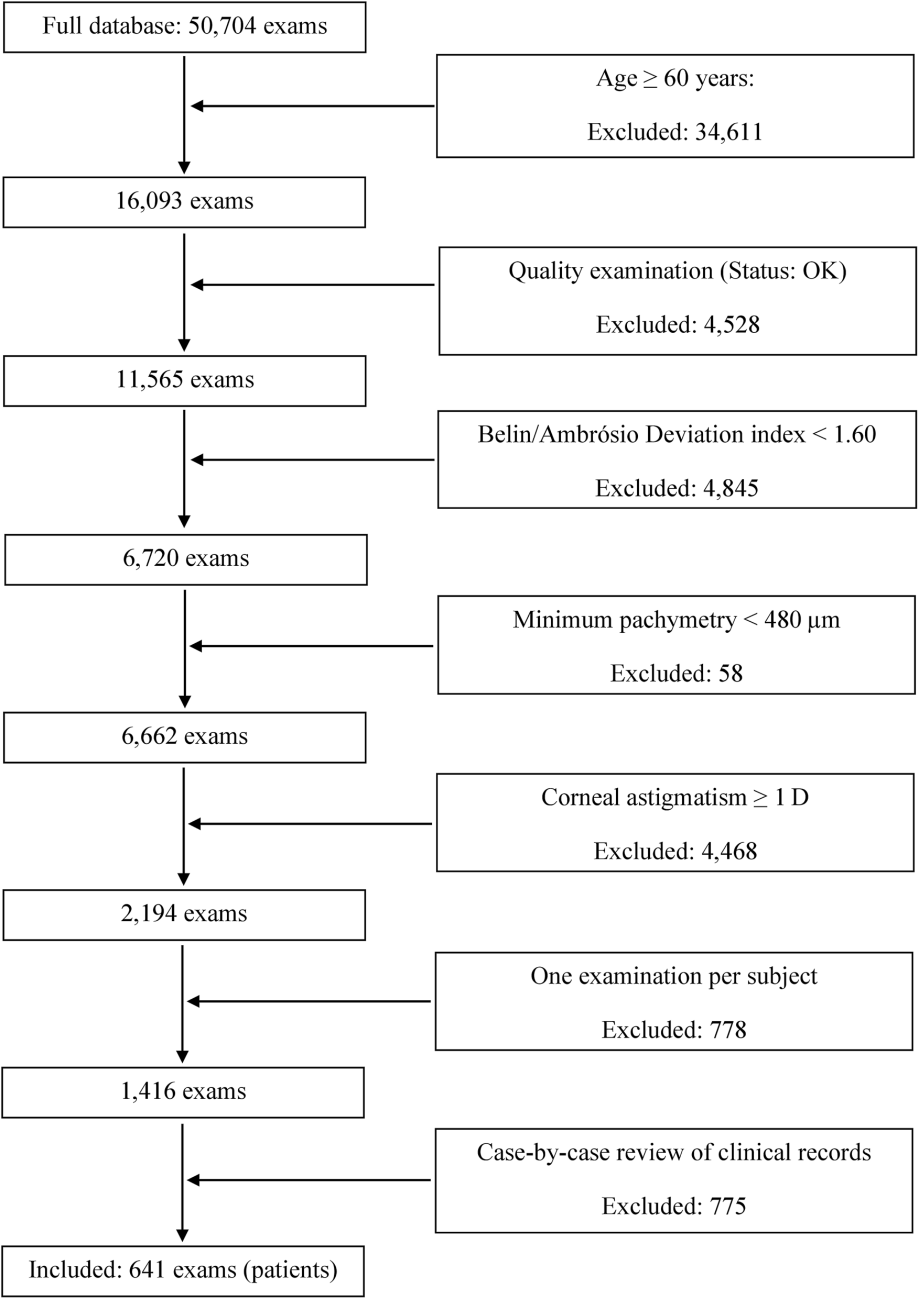
Flow diagram of the process of selection of cases from the patient-examination database.

We performed a retrospective analysis of corneal topography results obtained from a Scheimpflug device (Pentacam, Oculus Optikgeräte GmbH, Wetzlar, Germany). The tenets of the Declaration of Helsinki were followed, and approval was obtained from the ethics committee of the University of Heidelberg. All patients admitted to Heidelberg University Hospital sign an informed consent on the usage of their anonymized results for medical and scientific purposes. The data were collected between October 2004 and June 2019 during routine examinations. The database comprised topography results of 11,953 patients. All records were recalculated and exported using the latest version (1.21r59) of Pentacam software. The selection process is presented in Fig. 1. First, we identified patients who, at the time of examination, had been 60 years old or older. Then, examinations with an acceptable quality score (Q.S.: OK) were selected. Those cases were eligible whose total corneal astigmatism measured at 3 mm was ≥ 1 D; our criterion reflects the standard power-range toric IOL, which begins with a 1.0 D cylinder power at the cornea plane. Other inclusion criteria were the Belin/Ambrósio Deviation index (BAD D) < 1.60^16^, and a pachymetry of 480 µm or higher at the thinnest point^17^, which aimed to exclude post-laser surgery corneas or keratoconus cases. Only one exam was randomly chosen for further analysis. Thus, only one eye per patient was assessed. The Pentacam device provides good intra-subject reliability of anterior and posterior corneal curvature measurements as shown by Chen et al^18,19^. Although averaging three consecutive results may improve intersession repeatability, in this study, we did not perform the comparison between patients' visits. Hence, we relied on the inclusion of a single examination for each subject in the statistical analysis. The criterion-based selection was followed by a case-by-case review for corneal abnormality performed by an experienced clinician. By reviewing medical history, we ensured that none of the assigned cases had previous corneal procedures or pathology. In modern cataract surgery, postoperative refraction is typically stable within one month after the initial procedure^20^. Although currently mini- and micro-incision are routinely performed, in the past, 4–6 mm wounds were created, which yielded a longer healing process and a continuous astigmatic shift up to three months after surgery^21^. In the current study, we also included historical data. Thus, to ensure that postoperative astigmatism is stable in all included cases, pseudophakic eyes were only included if the time elapsed between cataract surgery and examination was at least three months^21^. Of 11,953 patients, 641 were deemed eligible.

The change of astigmatism with diameter was evaluated using corneal power distribution maps. The measurements were taken in five concentric zones from 2 to 6 mm (a 1-mm increment) positioned on the pupil center. The largest area corresponds to the scotopic pupil size and the optic diameter of a typical IOL^22^. True Net Power (TNP) parameter was assessed, which is a product of combined astigmatism from the anterior and posterior corneal surface. Corneal astigmatism was divided into three categories. With-the-rule astigmatism (WTR) was classified for the steep meridian between 60º and 120º, against-the-rule (ATR) for 0º–30º and 150º–180º; the remaining cases fell into an oblique-astigmatism category^23^. This astigmatism classification is often used in the field of cataract and refractive surgery. Please note, however, that in other studies, astigmatism can be expressed in a negative cylinder format, where WTR has an axis near the horizontal meridian, and ATR's axis lies near the vertical meridian. The other approach may refer to a spectacle correction of corneal astigmatism, which can also be used in astigmatism classification. Consequently, in this study, we applied a plus-cylinder approach, which corresponds directly to the recorded topography values. The assessment of the magnitude of astigmatism yielded further classification into low (< 1.75 D), moderate (between 1.75 D and 2.75 D), and high (> 2.75 D) astigmatism^24^.

Statistical analysis was performed using a Statistics and Machine Learning package (MathWorks, USA). Following correction for enantiomorphism, corneal astigmatism was converted to power vectors, as described by Thibos et al. using the following formula^25^:

$$J_{0} = - \frac{C}{2}cos\left( {2\beta } \right)$$

$$J_{45} = - \frac{C}{2}sin\left( {2\beta } \right)$$

where C is the difference between the flat and steep meridian, and β is the flat meridian. The J_0_ and J_45_ components were used to calculate the absolute difference in astigmatism measured at 2 mm, 3 mm, 4 mm, and 5 mm and the maximum diameter of 6 mm as the subtraction of the power vectors (e.g., J_0_@2 mm–J_0_@6 mm and J_45_@2 mm–J_45_@6 mm). The centroids (the magnitude of astigmatism × steep meridian in degrees) were calculated according to the formula:

$$Magnitude\;of\;astigmatism = 2\sqrt {J_{45}^{2} + J_{0}^{2} }$$

$$Axis = \frac{1}{2}tan^{ - 1} \left( {\frac{{J_{45} }}{{J_{0} }}} \right)$$

For negative J_0_, the axis was corrected by the addition of 90°. For positive J_0_ with a negative J_45_ component, a 180° correction was applied.

Normality was assessed using the Kolmogorov–Smirnov test and Q-Q plots. Corneal-astigmatism components proved non-normally distributed; thus, non-parametric statistical methods were chosen. Descriptive statistics included the median with the interquartile range (IQR).

In the clinical situation, the selection of a toric IOL is typically based on astigmatism measurements taken in the central cornea (between 2.5 and 3 mm)^26^. Thus, the absolute difference between 3 and 6 mm was used for statistical analysis. We applied a Friedman test to compare multiple groups and a Bonferroni post-hoc analysis. The results from left and right, as well as phakic and pseudophakic eyes, were compared with the Mann–Whitney U test. The Spearman rank correlation was used to calculate the association between the central and the mid-peripheral cornea.

## Results

The median age of the study population was 70.8 years (65.4–78.7 years). The right/left eye proportion was 45%/55%. Of the 641 eyes, 80 were pseudophakic. Low astigmatism had the highest prevalence in the study group (58%), 29% were in the moderate range, and 13% had astigmatism greater than 2.75 D. The determination of the type of astigmatism revealed that 53% of eyes had WTR, 34% had ATR, and 13% had oblique astigmatism. The astigmatism classification only slightly differed while evaluated at 6 mm (51% WTR, 37% ATR, and 12% oblique), indicating the lack of significant axis changes with corneal diameter.

**Figure 2:**
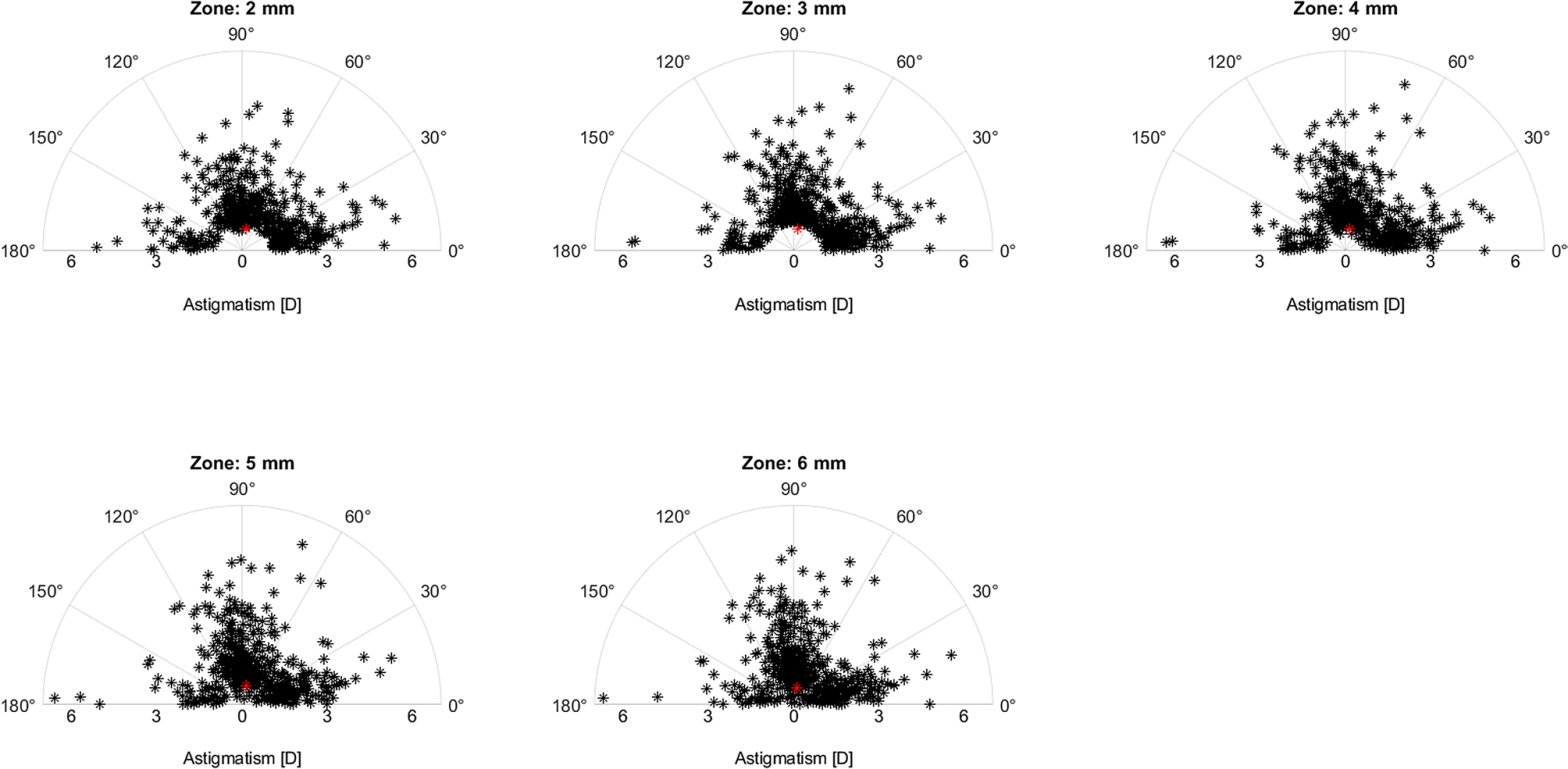
Single-angle polar plot of individual cornea astigmatism (black asterisk) of the study population. Red asterisks indicate the centroid for each corneal zone measured from 2 to 6 mm.

**Figure 3:**
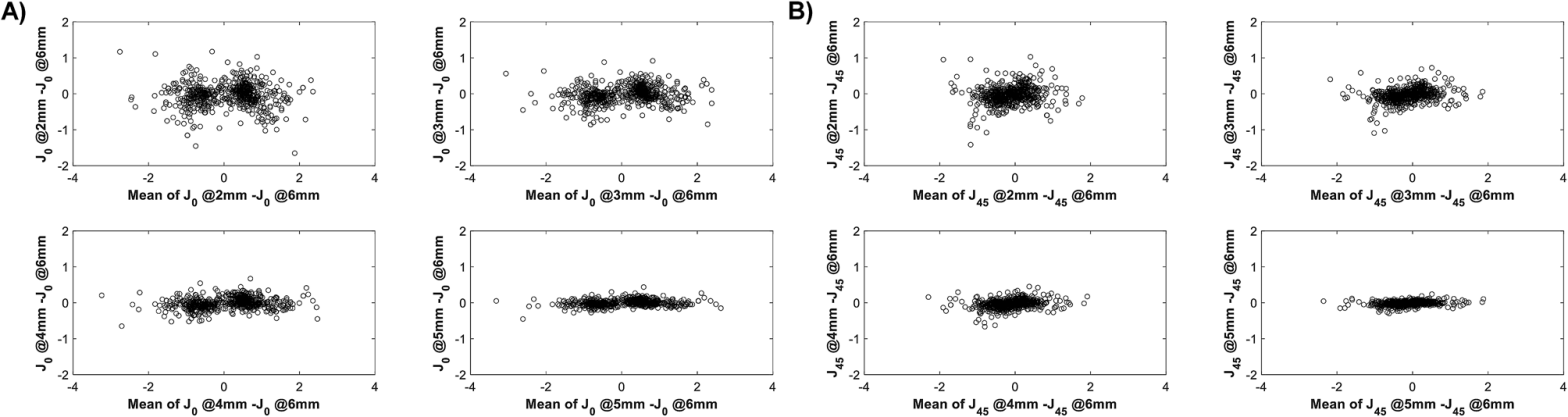
The subtraction of the J_0_ (**A**) and J_45_ (**B**) components measured at 6 mm and the other concentric areas (from 2 to 5 mm) versus the mean.

**Figure 4:**
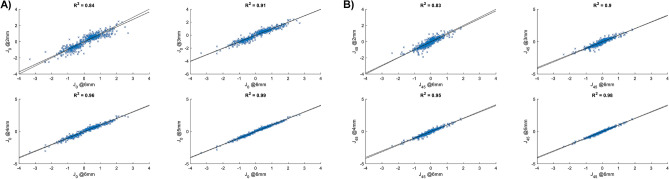
Intra-subject correlation of the J_0_ (**A**) and J_45_ (**B**) components of the power vector measured at 6 mm and the other concentric areas (from 2 to 5 mm). The black line refers to x = y; the dashed line is the linear regression model; R^2^ is the correlation coefficient.

Table 1 shows the characteristics of the studied corneas. Figure 2 shows the corneal astigmatism distribution in the study population. None of the study cases had the magnitude of astigmatism lower than 1 D at 3 mm. This creates a 'gap' in the polar plot of the central cornea (Fig. 2), which becomes less apparent at 6 mm due to decreasing TNP with diameter. The median absolute difference (IQR) between astigmatism measured at 2, 3, 4, 5 mm and the 6 mm area a was 0.30 D (− 0.36 to 0.64), 0.29 D (− 0.13 to 0.55), 0.20 D (− 0.10 to 0.38 ), 0.10 D (0 to 0.20), respectively. Figure 3 shows the differences between individual vector components. The comparison in the magnitude of astigmatism between the 2- and 6-mm zones yielded the larges deviation. However, this difference widened, while the cylinder power increased (Table 2). The analysis of the correlation coefficient between the power vectors measured at 6 mm, and the four areas confirmed the presence of differences between the central and the mid-peripheral (6 mm) cornea (Fig. 4).

**Table 1:** Description of corneal astigmatism measured in five concentric zones from 2 to 6 mm.

Zone:	2 mm	3 mm	4 mm	5 mm	6 mm		
K_Steep_–K_Flat_ [D]	1.6	1.7	1.6	1.6	1.5	*P*<.001	
IQR	1.3 to 2.2	1.3 to 2.3	1.3 to 2.2	1.2 to 2.2	1.1 to 2.2		
Steep meridian [°]	101.1	100.3	100.5	100.0	99.2	*P*=.97	
IQR	22.3 to 142.3	21.7 to 140.1	21.6 to 142.2	20.9 to 142.8	20.0 to 138.5		
J_45_ [D]	−0.15	−0.15	−0.13	−0.13	−0.11	*P*<.001	
IQR	−0.52 to 0.25	−0.52 to 0.24	−0.49 to 0.23	−0.45 to 0.21	−0.41 to 0.19		
J_0_ [D]	0.34	0.35	0.35	0.31	0.27	*P*<.001	
IQR	−0.62 to 0.68	−0.65 to 0.69	−0.64 to 0.69	−0.59 to 0.69	−0.54 to 0.67		

J_45_ and J_0_ are power-vector components (see Methods for more details). The *p* value was determined using the Friedman test for repeated measures comparison. K = keratometry; IQR = interquartile range.

**Table 2:** The comparison of the median absolute difference (IQR) in astigmatism for corneas with low, moderate, and high astigmatism.

	Low (<1.75 D)		Moderate (1.75–2.75 D		High (>2.75 D)	
2–6 mm	0.23 D	(−0.34 to 0.52)	0.37 D	(−0.42 to 0.80)	0.43 D	(−0.37 to 1.00)
3–6 mm	0.23 D	(−0.13 to 0.47)	0.35 D	(−0.07 to 0.62)	0.44 D	(0.00 to 0.90)
4–6 mm	0.14 D	(−0.10 to 0.32)	0.25 D	(−0.03 to 0.42)	0.30 D	(−0.11 to 0.61)
5–6 mm	0.10 D	(0.00 to 0.19)	0.11 D	(0.00 to 0.22)	0.13 D	(−0.10 to 0.30)

**Figure 5:**
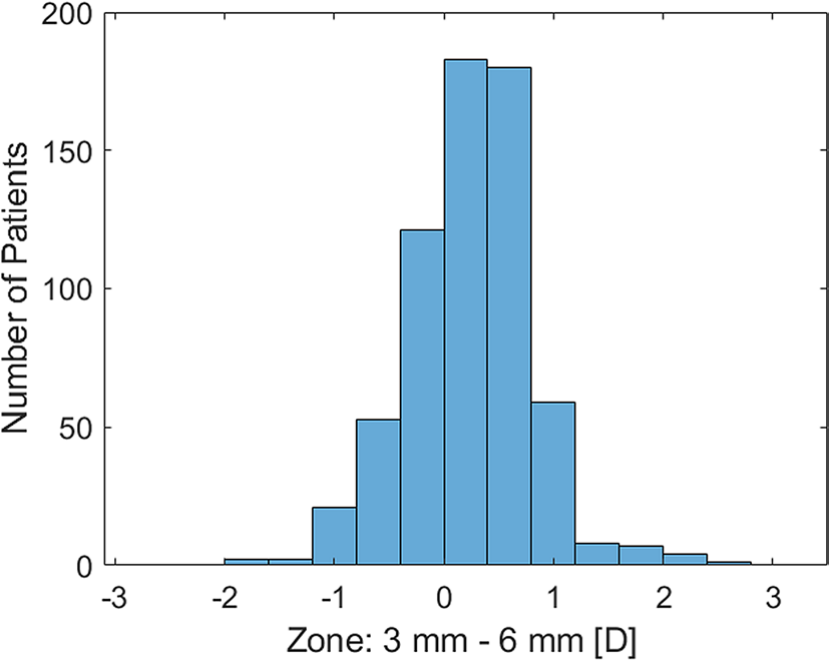
The distribution of the magnitude of astigmatism difference, calculated for each subject as the subtraction of astigmatism at 3 mm from that at 6 mm. Positive values indicate a reduction in corneal astigmatism in the mid-periphery. The corneas count with no change of corneal astigmatism was equally distributed between the first positive and negative bins.

Figure 5 presents the distribution of the absolute differences calculated as the subtraction of power vector components measured at 3 mm from that at 6 mm. Most of the study cases (66%) demonstrated a decrease of astigmatism with increasing corneal diameter, the reverse was found in 27%, and no change was noted in 7%. The statistical analysis of the absolute astigmatic difference between the 3- and 6-mm zone confirmed the similarity between left and right eyes (Mann–Whitney U test, *P* = 0.25), and phakic and pseudophakic eyes (Mann–Whitney U test, *P* = 0.43). However, the Kruskal–Wallis test revealed that eyes with WTR, ATR, and oblique astigmatism differ significantly (*P* < 0.001). The post-hoc analysis indicated that the decrease of mid-peripheral astigmatism is more substantial in eyes with ATR (*P* < 0.001) and oblique astigmatism (*P* = 0.002) than those with WTR (Table 3).

**Table 3:** Changes of the median absolute difference (IQR) in astigmatism found in three types of astigmatism.

	With-the-rule (WTR)		Against-the-rule (ATR)		Oblique	
2 –6 mm	0.16 D (−0.47 to 0.56)		0.36 D (−0.23 to 0.71)		0.40 D (−0.11 to 0.79)	
3 –6 mm	0.23 D (−0.28 to 0.49)		0.36 D (0.10 to 0.61)		0.39 D (0.00 to 0.64)	
4 –6 mm	0.12 D (−0.15 to 0.34)		0.23 D (0.00 to 0.42)		0.22 D (0.00 to 0.41)	
5 –6 mm	0.04 D (−0.10 to 0.20)		0.11 D (0.00 to 0.21)		0.10 D (0.00 to 0.20)	

## Discussion

We demonstrated that, in most patients (66%) in the elderly population, corneal astigmatism decreases from the center to the mid-periphery. However, the extent of this difference depends on the magnitude and the type of corneal astigmatism.

This decrease in astigmatism results from flattening of the steepest meridian as reported by Read et al^13^. The flattest meridian also progressively flattens towards the periphery, but to a smaller extent, which yields a decrease of the astigmatism magnitude^13^. They obtained topography data of the anterior cornea using a Placido disc video-keratoscope with a 0.5-mm annulus of varying diameter. Following the conversion to power vectors, they reported a decrease of the J_0_ component from 0.34 D at 2 mm to 0.28 D at 6 mm, which is close to the level that we found in the current study. The results are comparable despite the apparent differences in the inclusion criteria of both studies, including the magnitude of astigmatism. Read et al. studied young subjects (on average, 24 years old) with low astigmatism, and thus WTR had the highest prevalence in their population. In our study, 42% had either ATR (negative J_0_) or oblique (negative or positive J_0_) astigmatism, which may explain a relatively lower J_0_ value of our population despite the inclusion of eyes with higher astigmatism. In another analysis, Kawamorita et al. examined the corneal topography of 76 patients scheduled for cataract surgery^14^. Although their methodology has parallels with the one presented here, in that they also measured corneal topography using the Scheimpflug device, front sagittal K-Readings instead of TNP were used to calculate astigmatism changes with corneal diameter. Kawamorita et al. also found that astigmatism significantly decreases with the distance from the cornea center, and they demonstrated that the mid-peripheral flattening increases in patients with greater corneal astigmatism^14^. Thus, the difference between the steepest and flattest meridian in the 3-mm and 6-mm zones depends on the cylinder power^14^. Their findings are in agreement with those of the current paper, as we also demonstrated that patients with high astigmatism tend to show a decrease of mid-peripheral astigmatism at a greater rate (Table 2). The absolute difference in the magnitude between the 3 and 6-mm zones reported by Kawamorita et al. was, on average, 0.56 D, which was about twofold higher than what we found in this study. We identified two potential population-related factors that may explain this discrepancy. (1) Kawamorita's group studied Japanese patients whose topography data may differ significantly from data obtained from Caucasians^27^. Although, in the current study, ethnicity was not recorded at the time of examination, it is not unreasonable to expect that our population at a German university hospital was predominantly Caucasian. (2) Low-astigmatic cornea had the highest prevalence in our study, with a total magnitude of 1.70 D (Table 1) which was lower than 2.00 D reported by the Japanese group^14^. Thus, the overall difference found in this study may be smaller than one found by Kawamorita et al., given the association of higher astigmatism with increased flattening of the mid-peripheral meridians. Despite differences between our methodology and those of Read and Kawamorita, all three studies provide evidence for the decrease of astigmatism as corneal diameter increases from the center to the periphery; and this relationship exists regardless of age and ethnicity in the population^13,14^.

Cross-age studies on corneal topography in a normal population have shown a shift from WTR to ATR astigmatism with age^10,11^. Yet, we found that WTR astigmatism had the highest prevalence in our population of patients over 60 years old, and this is not an isolated finding, as a similar distribution of the three types of astigmatism was reported in a paper by Hoffmann and Hütz^28^. They studied biometry results of cataract patients, including corneal astigmatism, using an IOLMaster (Carl Zeiss Meditec, Germany). In that large-population study, 15,448 patients were included with the median age of 74 years. Hoffmann and Hütz reported that most of their patients had WTR astigmatism (46.8%), 34.4% had ATR, and 18.9% had oblique astigmatism. Those results are close to what we found, which may reflect similarities in populations as both studies were conducted in Germany. Correspondingly, Kawamorita et al. also reported the predominance of WTR over ATR astigmatism (63% vs. 37%) in Japanese subjects^14^. However, in that study, the classification of oblique astigmatism was omitted. They concluded that the type of astigmatism does not affect mid-peripheral TNP^14^. By contrast, we found that significant differences exist with the type of astigmatism being a differentiating factor.

In our population, the group with oblique astigmatism demonstrated a larger absolute difference between the central and mid-peripheral TNP; this was followed by the ATR-group, and then the WTR-astigmatic group had the smallest difference (Table 3). Although there is a difference in the magnitude, a decrease of mid-peripheral astigmatism was found in all three groups. There is evidence from Goto et al. on gender differences, particularly in relation to aging^29^. They found a higher prevalence of ATR astigmatism in male subjects, which was later confirmed by Hayashi et al^30^. Although we did not stratify our data based on subject's gender, as this information is not routinely collected by the Pentacam device, a stronger tendency for ATR astigmatism in men may suggest a higher difference in mid-peripheral astigmatism decrease between male and female patients. This conjecture, however, warrants further studies.

Ferrer-Blasco et al. reported that the prevalence of corneal astigmatism equal to or higher than 1 D in an elderly population is high and accounts for 34.8% of cataract patients^8^. In another large-cohort study, Day et al. showed that 42% of patients scheduled for cataract surgery have preoperative astigmatism of 1 D or greater^9^. Uncorrected corneal astigmatism may decrease the patient's unaided visual acuity and increase the need for additional spectacle correction for far vision after surgery^31^. This, in turn, can lead to patient dissatisfaction. Postoperative outcomes can, however, be improved as corneal astigmatism can be successfully corrected with implantation of a toric IOL^32^. Up until now, the toric IOLs have only been offered with a constant cylinder power throughout the IOL surface, which is suited for 7% of the population, according to the current study. In the paper by Kawamorita et al., this percentage was even lower, and that was 4%^14^. Although Read et al. reported the lack of differences between central (0–4 mm) and peripheral (4–8 mm) astigmatism in 51% of the studied corneas, this might have been expected, given a low level of corneal astigmatism (− 0.32 ± 0.6 D) in that population^13^. However, the variability between astigmatic corneas exists, as 17% of Japanese patients had an increase of mid-peripheral astigmatism^14^; we found 27% to share this pattern. On the other hand, in Kawamorita's and our study, the highest proportion of cases (79% and 66%, respectively) demonstrated a significant decrease of cylinder power with the distance from the cornea center. Thus, a toric design with progressively changing cylinder power with corneal diameter could mitigate the impact of the difference between central and mid-peripheral astigmatism. Unlike the magnitude of astigmatism, the axis and thus the type of astigmatism do not change at the periphery (Table 1)^14^. The production of a progressive toric IOL—with progressively less cylinder power toward the periphery—and their implementation in cataract surgery would appear to be worthwhile and desirable. Also, our comparison between phakic and pseudophakic eyes confirmed that cataract surgery does not affect this corneal property. However, such a novel approach would still require extensive evaluation in laboratory and clinical research to better understand its potential impact on the image quality and benefits to patients with stable, increasing, and decreasing mid-peripheral astigmatism.

Our focus is on the distribution of corneal characteristics in the population; thus, we did not assess the impact of mid-peripheral astigmatism on vision, which may be considered a limitation of our study. However, the effect on visual acuity of the reported astigmatic changes may still be evaluated using a simple model proposed by Blendowske^33^, which uses spherical and cylindrical components of the refractive error to predict unaided visual acuity in an otherwise healthy eye. According to this model, the population's change of 0.29 D in mid-peripheral astigmatism would not cause a consequential visual acuity loss. Assuming a 0 D spherical component, visual acuity at 6 mm pupil would be reduced by less than one letter of an Early Treatment of Diabetic Retinopathy Study chart (< 0.02 logarithm of the minimum angle of resolution [logMAR]). However, in the current study, we also observed a broad range of mid-peripheral corneal flattening and steepening (Fig. 5). In about 36% of the studied corneas, the astigmatism change falls between 0.4 and 0.8 D (both positive and negative). The Blendowske model predicts that in the absence of sphere, the cylinder of 0.7 D results in a one-line reduction of visual acuity, which may be considered clinically significant. Nearly 16% of the population demonstrated a more substantial corneal astigmatism change up to approx. 2.0 D. Such an extreme difference in astigmatism may severely impact patients' vision with a predicted visual acuity loss of 0.47 logMAR.

Further estimates on the impact of mid-peripheral astigmatism change in patients after toric-IOL implantation can be found in a study by Visser et al^15^. They described two cases with increased residual astigmatism after unremarkable toric-IOL implantation. Despite a correct alignment of the IOLs after implantation, one patient had astigmatism overcorrection by 1.75 D, as shown by manifest refraction^15^. In that case, corneal astigmatism measured at 6 mm was lower than that at 4 mm by 0.81 D. The patient had a decreased visual acuity of 20/30 (0.18 logMAR), which improved to 20/22 (0.04 logMAR) after a full spectacle correction. In the second described case, the patient's postoperative astigmatism was undercorrected with the refraction of + 0.25 D − 1.0 D × 102° yielding an unaided visual acuity of 20/50 (0.40 logMAR)^15^. However, after spectacle correction, full visual acuity was restored (i.e., 20/20 or 0.00 logMAR). Among other factors, the pupil-size related changes in corneal astigmatism were identified as the reason for increased residual astigmatism^15^. Visser et al. suggested measuring the pupil's diameter in a preoperative assessment of candidates for toric IOLs to flag patients at risk of this postoperative surprise^15^. In such cases, the implantation of an IOL that corrects mid-peripheral flattening or steepening may indeed prove advantageous. However, more research is needed to assess in optical-simulation and clinical studies how this progressive alternation of astigmatism affects patients' visual function.

In summary, we demonstrated that the magnitude of corneal astigmatism decreases with the distance from the center, but the axis of the principal meridian remains largely unchanged. Although most of the corneas in our study population showed a similar progressive pattern, patients with ATR and oblique-astigmatism, as well as those with astigmatism higher than 1.75 D, are most affected by this mid-peripheral change in corneal astigmatism.

## Data Availability

The datasets generated during and/or analyzed during the current study are available from the corresponding author on request.
